# Photographic Illustrations of Skin Diseases

**Published:** 1889-11

**Authors:** 


**Photographic Illustrations of Skin Diseases—An Atlas and Text-Book combined. Second Series, complete in twelve parts.** By GEORGE HENRY FOX, A. M., M. D., Clinical Professor of Diseases of the Skin, College of Physicians and Surgeons, New York. Hand-colored plates; nearly 100 cases from life. New York: E. B. Treat, No. 771 Broadway. New York: Parts IX. and X. Price, $2.00 each.

Part IX. concludes the article on Hypertrichosis from Part VIII. of this series, and then furnishes an excellent plate of hypertrophy of the nails. The atrophic diseases are then taken up, and Albinismus, Leucoderma Canities, and Alopecia are treated. Chapter VI. is devoted to the consideration of neoplastic diseases, as Cicatrix, Keloid, and Fibroma, the latter being continued in Part X., which also treats of Xanthoma, Neuroma, Telangiectasis, Nevus Vasculosus, Angioma, Lupus Vulgaris and Erythematosus, Scrofuloderma, and Syphilis.

The plates are good illustrations of the diseases of which the text treats, and afford a clearer insight into this obscure and difficult subject than could be obtained by the physician in any other way.

We think the author is performing a useful service to the profession in this work, and we commend it as meritorious and valuable. The plates and press-work are unexcelled.

